# The Sixth Year Molar

**Published:** 1920-02

**Authors:** John E. Gurley

**Affiliations:** San Francisco


### THE SIXTH YEAR MOLAR

JOHN E. GURLEY, SAN FRANCISCO

The sixth year molar—the most important tooth—why? Characteristically different from all of the other teeth—how? It is different from all of the other teeth in that:

1. It is the only permanent tooth whose development is similar to that of the deciduous teeth, namely, that its enamel organ springs directly from the dental lamina.

2. It is the only permanent tooth whose crown is completely calcified by the time "the individual is thrown upon his own resources for obtaining nourishment.' (Noyes.)

It is the most important tooth in that:

1. It is the first or at least one of the first of the permanent teeth to erupt, and in too many cases is mistaken by the parent for a deciduous tooth, and consequently, on account of lack of attention, is subject to caries.

2. It is erupted just prior to the loss of the deciduous molars, a time when developmental changes are most active in the jaws, and thus at once becomes:

3. The key to occlusion, by interlocking with its fellow of the opposite jaw, and holding these corresponding points in the two jaws, in the same relative position until such time as full replacement shall have taken place.

### HISTOLOGY AND DEVELOPMENT

Immediately posterior to the enamel organs of the deciduous teeth, at about the seventeenth week of foetal life, the development of the enamel organ of the first permanent molar is begun, and by the ninth month the follicle is complete and calcification has begun. This means that at birth a line of the cusps is calcified. This calcification continues regularly throughout the first few years, until, at the age of about five years, the crown is completely calcified. At about six to seven years the tooth is erupted, the roots being only about one-third developed.

### ANATOMY

A study of the anatomy of the molar teeth reveals a difference between the upper and lower molars—the general shape of the upper being that of a rhomboid, while the lower is similar to a trapezoid.

Both present cusps, ridges and grooves, which in this consideration we shall not discuss in detail. Uppers and lowers differ, both as individual groups, and also as members of each group, in size, prominence of cusp, and depth of groove. Usually the lower molar presents the deepest groove, while the upper may have the most prominent cusps, with deep mesial and distal pits. Large teeth generally present the deepest grooves or fissures. Black gives, as a reason for this, the suggestion that "the growth of the dentin pulp in large teeth is often too great for the enamel cap, so that the enamel-forming cells are drawn apart, preventing a union of the enamel plates." The principal grooves are the so-called buccal grooves of both upper and lower, and the lingual groove of the upper molar. There is no lingual groove to be found on the lower molar, and as Cryer points out, caries is seldom found on this surface.

### CARIES

Marshall gives, to my mind, the most comprehensive statement that I have seen:

"Dental caries is a progressive molecular disintegration of the structural elements of the tooth, beginning with the solution of the inorganic substances by the action of lactic acid formed within the mouth by fermentation, and terminating with the dissolution of the organic matrix through the solvent action of the saprophytic micro-organisms."

This requires that we give some attention to food and fermentative changes that may take place within the mouth. Foods may be classified as follows:

1. Proteids—tissue builders.
   - (a) Animal—nitrogenous, do not undergo fermentation, but putrefaction.
   - (b) Vegetable.
2. Fats—fat and heat producing.
3. Water.
4. Salts.
5. Carbohydrates—energy and fat producing.
   - (a) Starches.
   - (b) Sugars.

The last named is the particular variety of foodstuffs which undergoes fermentation, the reactions **being** indicated as follows:

1. C6 H12 06 (Fruit Sugar) equals 2(C3 H6 03) (Lactic Acid).
2. C12 H22 011 (Cane Sugar) plus H2 0 equals C6 H12 06 (Dextrose) plus C6 H12 06, C6 H12 06 equals 2(C3 H6 03) (Levulose).
3. (C6 H10 05 plus H20)n (Starch) equals (C6 H10 05)n (Dextrin) plus (C12 H22 011)n C12 H22 011 (Maltrose) plus H20 equals 2(C6 H12 06) (Glucose).

   C6 H12 06 equals 2(C H6 03) (Lactic Acid).

The first two of these are mono- and di- saccharides, respectively, the mono- being the simplest form of sugar. Disaccharides can, by hydrolysis, be made to yield two molecules of the mono- type. Poly-saccharides, or starches, have unknown molecular weight and will yield an unknown number of molecules of mono-saccharide when completely hydrolized. As indicated in 2 and 3 above, hydrolysis first takes place, following which the simple sugar is split into molecules of lactic acid.

Simple sugars are sure to be found in candy, honey, etc., while potato is largely starch, which may be reduced to simple sugar. It is at once conceivable, then, that any such remaining within the mouth, about the teeth, may be converted into lactic acid, with its destroying effects, as mentioned in our definition.

Now, then, let us examine the mouth of a child at the age of seven years up. The incisors are being replaced; the sixth year molars are in process of eruption, lying below the occlusal line of the deciduous teeth, and are quite inaccessible to ordinary means of cleansing; the fissures and grooves are deep; food packs therein; the tooth is half-covered by gum tissue, and is protected by the second deciduous molars and this gum tissue. The gum tissue about the crowns is loose and will continue to be until the tooth is fully erupted. Consequently, food packs under the free margin and is held firmly in place. What is the result? Caries in the buccal groove and on the occlusal surface. Also as the molar pushes through into its place, there is more often than not the possibility that food may be lodged and held between it and the second deciduous molar, resulting in the formation of an approximal cavity.

Caries rarely occurs on the lingual surface of lower molars, but when present, I believe that it is due to a lingual tipping of that tooth, which affords protection and precludes access with the toothbrush. Caries of the lingual surface of upper molars is more common, though generally confined to the lingual groove, and is most likely the result of extension from the occlusal surface out. Occasionally caries may occur about the supplemental cusp, found near the mesio-lingual cusp, and which may be explained as due to the protection afforded, or inaccessibility in the matter of cleansing.

This does not mean that we shall preclude sugar or rather carbohydrate from the child's diet; in fact, carbohydrate food is very essential—it is the energy producer, and therefore highly necessary. But it does mean that too great care in the matter of prophylaxis can not be exercised.

Dr. Harvey W. Wiley says that he'd like to be food director of this country for awhile; that he'd close all the candy shops, not only for the period of the war, but for all time. He says that a child gets all the carbohydrate food he needs in a well-arranged diet, and that no person under fifteen should be given sugar in any other form than that found in his regular menu.

Look, now, to the child on above seven—the deciduous molars are being replaced; note the spaces, pockets, etc., cavities, in some of the teeth; you men who say that "there is nothing to do for this child," calculate these pockets and contemplate the vast amount of fermenting and putrifying food to be found here. Is it any wonder that we find more caries?

But confining ourselves to the sixth year molar, we find just cause for cavities on all surfaces. What can we do with those now there, and what can we do to prevent those that are not there from making an appearance?

### TREATMENT

Treatment of these conditions demand the use of wisdom and judgment. Many things must be taken into consideration—the type of child, whether timid or bold, the age, extent of decay, possibility of recurrence, natural cleansing ability, etc.

Generally speaking, buccal, lingual or approximal cavities may be filled with amalgam, though oftentimes the use of cement is to be preferred. If the buccal groove is deep, recurrence is quite likely, and there is a good reason for not cutting well up into the groove, use cement. Approximally, the same suggestion applies, particularly in case of a deep cavity, or if the marginal ridge be involved and there is good reason for not preparing a suitable cavity. And I believe that there is a good reason for not preparing a suitable cavity if the child be under ten years and the work painful. I use practically nothing but cement in occlusal cavities in children under ten to twelve years—I do not like to cut extensively into the undeveloped teeth, nor do I care to put the child through too extensive operations. Metal fillings are likely to be lost, anyway, so better lose the cement. In dealing with children, we should take care of them, rather than attempt such as we call ''permanent operations'' for the adult.

There is yet one other condition, or rather a more extensive condition, namely, the progress of decay, generally occlusally, until the pulp is almost reached. We take out layer after layer of decayed dentin, until finally we know that to remove just one more layer will expose the pulp. Here we must stop, and by the following treatment we can, in the great majority of cases, check the decay and thus save the vitality of the tooth. After having reached the last point in excavation, where at least comparative safety exists, sterilize with ten per cent. silver nitrate solution, cover with Ames Pulp capping material, then complete with the ordinary copper cement, and you will be surprised to note how many pulps can thus be saved. Or, if you prefer, you may use Howe's silver nitrate method, which consists of precipitating metallic silver from an ammoniacal solution of silver nitrate by formalin or preferably eugenol. Care must be exercised in the use of formalin, however, or destruction of the pulp will ensue.

I am strongly of the opinion that synthetic cements should not be used in children's teeth, and especially in deep cavities; they are pulp destroyers. Incidentally, these have a very limited field of use, to my mind, in very shallow cavities, or over a heavy layer of cement in the anterior teeth of adults.

However, in the consideration of the question of the sixth year molar and its permanent retention, the superficial decay is of secondary importance. These cavities can be successfully handled in any one of a number of ways, provided the operator is determined. The really serious question is the relation of decayed dentin to the pulp. If it has progressed until the pulp is exposed, we find ourselves face to face with a difficult problem. We at once realize the importance of this tooth—the root is as yet undeveloped and can not be filled; there is but one recourse, according to Black, and that is to remove the tooth. In fact, Black is of the opinion that a molar of a child under fourteen or fifteen, which has the root canals filled, will not last until the person is thirty years old.

In case repair is impossible, and extraction is to be resorted to, it may be possible to render some sort of temporary repair, so that a suitable time for removal may be chosen. By this I refer to the age or some other condition—for, all things considered, the best arrangement of the features and the remaining teeth will be obtained if they are removed about the ninth or tenth year. If the child be older than this at the time of the first visit, it may be possible to consult an orthodontist, as has been my experience, in which cases we have filled the canals and thus retained the teeth for a definite purpose and a limited time.

### PREVENTION

Preventive dentistry and preventive medicine are in the minds of men more to-day than at any time in history —it is realized more keenly than ever before that preventive measures must be taken in the matter of disease. What can we do in the practice of preventive dentistry so far as the sixth year molar is concerned?

1. Use every possible endeavor to get hold of the child under six years of age.
    (a) This is easily done in private practice—simply ask the mother for the child.
    (b) By the establishment of school and institutional clinics.
    (c) Awaken the dentist to a realization of his responsibilities.
2. Show the mother the value of the sixth year molar.
3. Give the mother and child definite instruction in:
    (a) Use of the brush.
    (b) Why, when and how to use it.
    (c) Recommend a good dentifrice.
    (d) Time of eruption of permanent teeth.
4. Do not allow food to pack under the free margin of the gum about the gingival part of the tooth.
5. Keep the sulci of the occlusal surfaces free from decomposing food—this may be easily accomplished by keeping these same sulci filled with Ame's copper cement, after having been thoroughly cleansed and sterilized.

6. See the child often and regularly for prophylactic and whatever repair work may be necessary.

By conscientiously carrying out the above, there can be little doubt as to the possibility of permanently retaining these most important molar teeth.

Feeding the child is of the utmost importance—regularity and kind must be religiously observed. Bunting has summed up the situation very clearly. He says: "As the deciduous teeth are lost, food has a tendency to pocket in the irregularities, oral fermentations are high, and caries common. The enamel of newly-erupted teeth is imperfect and less resistant than at a later period. The period of childhood is the susceptible period and the time when effective measures of caries control must be employed. We must not wait until the little patient has become an adult and thus more satisfactory to us. Children must be seen regularly; assist and encourage them to keep their mouths clean; fill cavities in their incipiency; orthodontic interference should be given when indicated; advise the use of hard foods with a limited amount of sweets, combined with tart and acid fruits; advise foods with a high calcium content, such as milk, buttermilk, cheese, celery, spinach, turnips, radishes, string and kidney beans, cabbage and cauliflower, and finally, the frequent use of limewater as a mouthwash."

It is a simple operation to extract a tooth, and only mechanical genius is needed in the construction of prosthetic appliances; but it is far more worth while when we have successfully warded off the enemies of that which nature intended for our permanent use. Give the child a chance, do what you can with the deciduous teeth; but look well to the sixth year molar, contemplate its importance, then look to its life, and great will be your reward. —*Journal of California State Dental Association* and *Northwest Journal of Dentistry*.

